# Scientific objectives and payload configuration of the Chang'E-7 mission

**DOI:** 10.1093/nsr/nwad329

**Published:** 2023-12-29

**Authors:** Chi Wang, Yingzhuo Jia, Changbin Xue, Yangting Lin, Jianzhong Liu, Xiaohui Fu, Lin Xu, Yun Huang, Yufen Zhao, Yigang Xu, Rui Gao, Yong Wei, Yuhua Tang, Dengyun Yu, Yongliao Zou

**Affiliations:** State Key Laboratory of Space Weather, National Space Science Center, Chinese Academy of Sciences, Beijing 100190, China; National Space Science Center, Chinese Academy of Sciences, Beijing 100190, China; National Space Science Center, Chinese Academy of Sciences, Beijing 100190, China; National Space Science Center, Chinese Academy of Sciences, Beijing 100190, China; Institute of Geology and Geophysics, Chinese Academy of Sciences, Beijing 100029, China; Institute of Geochemistry, Chinese Academy of Sciences, Guiyang 550081, China; Shandong Provincial Key Laboratory of Optical Astronomy and Solar-Terrestrial Environment, School of Space Science and Physics, Institute of Space Sciences, Shandong University, Weihai 264209, China; National Space Science Center, Chinese Academy of Sciences, Beijing 100190, China; National Space Science Center, Chinese Academy of Sciences, Beijing 100190, China; Institute of Drug Discovery Technology, Ningbo University, Ningbo 315211, China; State Key Laboratory of Isotope Geochemistry, Guangzhou Institute of Geochemistry, Chinese Academy of Sciences, Guangzhou 510640, China; School of Earth Science and Engineering, Sun Yat-sen University, Guangzhou 510275, China; Institute of Geology and Geophysics, Chinese Academy of Sciences, Beijing 100029, China; Lunar Exploration and Space Engineering Center, Beijing 100190, China; China Aerospace Science and Technology Corporation, Beijing 100048, China; State Key Laboratory of Space Weather, National Space Science Center, Chinese Academy of Sciences, Beijing 100190, China; National Space Science Center, Chinese Academy of Sciences, Beijing 100190, China

**Keywords:** Chang'E-7, scientific objectives, scientific payloads

## Abstract

As the cornerstone mission of the fourth phase of the Chinese Lunar Exploration Program, Chang'E-7 (CE-7) was officially approved, and implementation started in 2022, including a main probe and a communication relay satellite. The main probe, consisting of an orbiter, a lander, a rover and a mini-flying probe, is scheduled to be launched in 2026. The lander will land on Shackleton crater's illuminated rim near the lunar south pole, along with the rover and mini-flying probe. The relay satellite (named Queqiao-2) will be launched in February 2024 as an independent mission to support relay communication during scientific exploration undertaken by Chang’E-4, the upcoming Chang’E-6 in 2024 and subsequent lunar missions. The CE-7 mission is mainly aimed at scientific and resource exploration of the lunar south pole. We present CE-7’s scientific objectives, the scientific payloads configuration and the main functions for each scientific payload with its key technical specifications.

## Teaser

This review provides an overview on the scientific tasks of Chang'E-7 mission in the fourth phase of China's Lunar Exploration Project, puts forward scientific objectives based on key scientific issues in lunar exploration focusing on the south pole, conducts the analysis of scientific requirements, and completes the system design of the scientific payload.

## INTRODUCTION

The Moon is the closest celestial body to Earth and has always been the first priority for human space exploration. In the late 1950s, with the launch of the Soviet Union's first lunar probe, an upsurge in deep-space exploration with the Moon as the main target occurred. In particular, the successful implementation of the Apollo program from 1961 to 1972 greatly enriched our understanding of the Moon and helped establish the theoretical framework of the Moon's formation and evolution, leading to the foundation of planetary sciences. In the 1990s, a new era of lunar exploration missions started again. Many countries and organizations, such as Japan, the European Space Agency (ESA) and India initiated their lunar exploration programs, in addition to traditional space-faring countries such as the USA, Russia and China. Since its beginning in 2004, the Chinese Lunar Exploration Program (CLEP) has been strategically composed of three phases: ‘Orbiting phase (Phase I), Landing phase (Phase II) and Sample Return phase (Phase III)’ [1,2]. In 2007, the first lunar probe of China, Chang'E-1 (CE-1), was successfully launched and has achieved important results; for example, it completed China's first full map of the Moon's surface with a spatial resolution of 12 m [3]. In 2010, Chang'E-2 (CE-2) carried out a detailed survey of Sinus Iridum—the potential landing site of Chang'E-3 (CE-3)—with a spatial resolution as high as 1.5 m, and conducted a fly-by observation of asteroid 4179 Toutatis [4]. In 2013, CE-3 successfully achieved a soft landing on the Moon and conducted a roving exploration in Sinus Iridum. It made significant progress in mapping surface mineral and chemical compositions, investigating substructure characteristics and evaluating geological evolution in the landing and roving areas [5,6]. In 2019, Chang'E-4 (CE-4) achieved a soft landing and roving exploration on the far side of the Moon for the first time in human history and made a series of achievements in studying the surface composition, subsurface structure, geological history and particle environment on the floor of Von Kármán crater in the South Pole-Aitken (SPA) basin [6–9]. In 2020, Chang'E-5 (CE-5) successfully collected 1731 g of lunar samples from Oceanus Procellarum and returned them to Earth, marking the completion of China's three-step lunar-exploration-mission goals of ‘orbiting’, ‘landing’ and ‘returning samples’ [10,11]. By carefully studying the returned lunar samples, significant scientific progress has been made in evaluating the geochronology of magmatic activity, the water-depleted lunar mantle source and the space weathering characteristics of lunar soils at the landing site [12–22]. Especially notable is that a new mineral, named Changesite-(Y), has been discovered [23]. With the implementation of the CLEP, multiple key technological breakthroughs have been made, such as orbiting, landing, roving, *in-situ* drilling, ascent from the lunar surface, rendezvous and docking in lunar orbit, and re-entry into the Earth's atmosphere. The original scientific and technological achievements have greatly promoted the development of lunar and deep-space exploration activities in China [24–26].

In January 2022, the State Council Information Office of China released a white paper entitled ‘China's Space Program: A 2021 Perspective’. The paper stated that in the next 5 years, China will continue to implement the fourth phase of the CLEP by launching the Chang'E-6 (CE-6) and Chang'E-7 (CE-7) missions. CE-6 will collect and bring back samples from the far side of the Moon, while CE-7 will perform a high-precision landing in the lunar polar region and undertake shadowed crater exploration. Finally, by completing research and development on the key technology of Chang’E-8 (CE-8) and in collaboration with other countries, international organizations and partners, a prototype of the International Lunar Research Station (ILRS) will be built. With the CLEP, China will carry out a comprehensive exploration of lunar geology and shallow subsurface structures, and strive to achieve significant advances in dating lunar magmatic activity, constraining mineralogical characteristics and analyzing the chemical compositions of the Moon [27].

CE-7 is a new type of probe developed for the fourth phase of the CLEP. It will explore the environment and resources in the lunar polar region by conducting a series of steps: orbiting, landing, roving and mini-flying. CE-7 includes the main probe and a relay satellite (named Queqiao-2). The relay satellite will be launched independently in early 2024 to support relay communication between the ground stations on Earth and CE-4, CE-6 and the subsequent CE-7 and CE-8 missions near the lunar polar regions, as well as deep-space very long baseline interferometry (VLBI) measurements and radio observations. The main probe consists of an orbiter, a lander, a rover and a mini-flying probe. It is scheduled to be launched by 5 March 2026 from the Wenchang Satellite Launch Site, located on Hainan Island. The orbiter and the relay satellite will establish a relay communication link between the lunar polar region and ground stations. The orbiter will use self-carried payloads to conduct a high-precision detailed survey of the landing area for ∼2 Earth months. After completion of the survey, the lander, rover and mini-flying probe will land on the lunar surface and carry out scientific investigations.

After briefly introducing international lunar exploration activities, we will present the CE-7 scientific requirements and discuss landing site selection, scientific objectives, payload configuration and technology specifications, respectively.

## INTERNATIONAL LUNAR EXPLORATION ACTIVITIES

Since the 1950s, multiple lunar exploration activities have been implemented. Remote observations, *in-situ* measurements and returned-lunar-sample analysis have led to a great understanding of the origin and evolution of the Moon and the solar system, promoting the rapid development of space science, space technology and applications. In the 21st century, due to its important scientific significance and the potential value of its resources, the Moon remains the first choice and key exploration target for countries worldwide to start their deep-space exploration activities; meanwhile, the Moon is also regarded as an ideal outpost for humans to enter deep space in the future [28–30]. The USA, Russia, Europe, Japan, India and other countries have released medium- and long-term plans or related strategies and policy documents for lunar exploration, with special emphasis on the important role of scientific goals in lunar and deep-space exploration missions.

Led by the USA, the Artemis program is currently being implemented and involves multinational efforts [31–33]. In 2022, the Artemis 1 mission used the new-generation American heavy-lift expendable launch vehicle Space Launch System to successfully launch the Orion spacecraft and 10 CubeSats into lunar orbit. The unmanned Orion spacecraft orbited around the Moon and returned to Earth, laying a solid foundation for future manned lunar exploration. The 10 CubeSats made multiple point observations of the Earth–Moon radiation environment [31]. According to the released plan, the crewed Artemis program aims to send astronauts back to the Moon in 2025, to build an Artemis Base Camp between 2025 and 2030 to achieve a long-term presence on the Moon and sustained lunar exploration and finally lay the foundation for future manned Mars exploration. In this program, the National Aeronautics and Space Administration (NASA) emphasizes collaboration with commercial partners to implement Commercial Lunar Payload Services (CLPS) and support small satellites for payload technology verification and resource exploration [31–33]. As of June 2023, 25 countries and regions had joined the Artemis Accords. In addition, in order to support manned Moon landings and future manned Mars exploration, NASA, together with ESA, the Japan Aerospace Exploration Agency (JAXA) and the Canadian Space Agency (CSA), proposed the Gateway lunar space station program. From 2024 to 2031, a comprehensive space hub integrating residence, experiments and energy storage will be gradually built. The Gateway will be the first space station beyond low Earth orbit and the first space station to orbit the Moon as well.

In April 2022, the US National Research Council released a decadal survey report named ‘Origins, Worlds, and Life: A Decadal Strategy for Planetary Science and Astrobiology 2023–2032’ [34]. The report states that in the next 10 years the USA will focus on several scientific priority fields and potential opportunities, such as planetary science, astrobiology and planetary defense; corresponding recommendations are also made [34]. The report identifies 3 high-level science themes, and defines 12 priority science questions to help guide mission selection and research in planetary science and astrobiology. It also suggests carrying out some relatively smaller-scale space missions, such as the lunar sample-return mission Endurance-A [34].

ESA put forward the idea of a ‘moon village’ in 2015. In July 2022, ESA released a new version of the space exploration roadmap ‘Terrae Novae 2030+ strategy roadmap’. The roadmap presents ESA's long-term exploration vision for human and robotic sustained space exploration activities in low-Earth orbit and on the Moon and Mars, maximizing the synergy among the three. ESA proposes new candidate missions and the technologies needed for the Terrae Novae exploration program within the framework of 2025–2030 and looks forward to sending the first European astronaut to the Moon before 2030.

Russia has prioritized lunar exploration as one of its development directions and is eager to quickly restore its former position as a major aerospace power through lunar exploration. It aims to boost national self-confidence through the implementation of the Luna 25 to Luna 28 missions. Unfortunately, the landing of Luna 25 failed, which dealt an enormous blow to the Russian aerospace industry. It is difficult to predict whether the subsequent missions of Luna 26, Luna 27 and Luna 28 can be carried out as scheduled, but it is estimated that cooperation with China at the International Lunar Research Station will be affected to some extent.

On 23 August 2023, India's Chandrayaan-3 successfully landed in the southern region of the Moon at 69.37°S and 32.35°E, making India the fourth country to successfully land on the Moon. Overall, the Chandrayaan-3 mission fully embodies the features of India's deep-space exploration—it dares to face the challenge of soft landing on the ‘no man's land’ of the lunar southern high latitudes based on its own characteristics. The scale of the exploration mission is realistic, short and concise, and the scientific exploration targets are specific and clear. Through international cooperation, any shortcomings in engineering are compensated and international influence is continuously enhanced.

Overall, in addition to NASA's plan of returning to the Moon, China, Russia, Japan, India, the United Arab Emirates and other countries are also implementing lunar exploration programs. A renaissance and enthusiasm for lunar exploration have once again been initiated [35]. Building lunar bases or lunar villages or scientific research stations near the polar regions on the Moon is becoming the main goal of future development.

## SCIENTIFIC REQUIREMENTS OF CE-7

The CE-7 mission will land near the lunar south pole, at the rim of the largest and oldest basin recognized on the Moon, i.e. the SPA basin, and in the vicinity of the permanently shadowed regions (PSRs) of the south pole [24]. From the perspective of the future location of the lunar research station, both the lunar north and south poles have good illumination conditions, and there may be water ice in the PSRs below the floor surface of adjacent impact craters. The lunar south pole region is located at the rim of the SPA basin, and it is a unique region for studying the early impact history of the Moon and the composition and structure of the Moon's interior [36–40]. Taking into account the future location of the lunar research station, the CE-7 mission will select the lunar south pole region as its preferred landing area.

**Figure 1.**
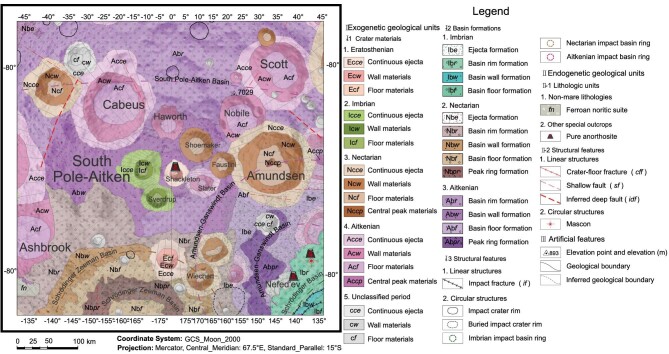
1:2 500 000-scale geologic map of the lunar south pole region [41].

The lunar south pole region is a highland terrain on the edge of the SPA basin. The terrain shows large elevation variations, mainly due to the excavation and ejecta deposition on the lunar surface by large-scale impact events. On the most recent geologic map of the Moon [41], the south pole region is predominantly characterized by various geologic units associated with craters and basins, including SPA, Amundsen-Ganswindt, Schrödinger-Zeenman, Schrödinger, Cabeus, Scott and Amundsen (Fig. 1). The SPA basin is believed to be the oldest impact structure in this region, followed by the formation of the Amundsen-Ganswindt basin, Scott crater, Cabeus crater and other Aitkenian craters whose terrains have been severely degraded. During the Nectarian Period, the Schrödinger-Zeenman basin, Amundsen crater and other contemporaneous craters formed, and these craters are better preserved than the older Aitkenian craters. One of the youngest lunar basins, the Schrödinger basin, also formed near this region, and its ejecta are widely distributed in the lunar south polar region. In addition, a small number of young impact craters formed during the Imbrian, Eratosthenian and Copernican period in this area, such as the Shackleton crater covering the lunar south pole.

On the periphery of the SPA basin, the main lithologic units in the lunar south pole are the ferroan anorthositic suite [42]. These ferroan anorthosites are plagioclase cumulate rocks with coexisting ferroan (Mg# ≤ 71) mafic minerals that occur in feldspathic highlands (FeO ≤ 10 wt%). Additionally, some magnesian anorthositic suite units are scattered sporadically among the ferroan noritic suite lithologic units. This mafic plagioclase is characterized by anorthositic rocks with magnesian (Mg# > 71) coexisting mafic minerals that occurred in feldspathic highlands (FeO ≤ 10 wt%). Simultaneously, there are some ferroan noritic suites, which are ferroan (FeO > 10 wt%, Mg# ≤ 71) and low-Ca pyroxene-rich non-mare rocks exposed on the SPA floor [43]. These different rock types are likely to represent the compositions of the original upper part of the lunar crust that is mainly composed of anorthosite that was excavated and redistributed during the formation of the SPA basin, as well as the exposed lower part of the lunar crust that is mainly composed of norite. Lava plains formed by large-scale volcanism and other volcanic landforms, such as lunar rilles, wrinkle ridges, domes or hypabyssal intrusive magma related to floor-fractured craters, have been reported in the SPA basin [44,45]. Nevertheless, these features are absent within the lunar south circumpolar region. However, there are some other structures: (i) the endogenic structures, which include deep faults, shallow faults, crater-floor fractures and mascon; and (ii) exogenic structures, which include impact fractures [46].

Due to the low illumination conditions in the polar region, there are many PSRs in the lunar south pole region [47]. The PSRs have maintained a low-temperature environment for a long phase of geological history, and there are likely to be volatile substances such as water ice that cannot exist stably in other areas of the lunar surface. The neutron detection data from the Lunar Prospector indicated a high H content relating to the PSRs [48]. Lunar Crater Observation and Sensing Satellite (LCROSS), Chandrayaan-1 and other probes have also carried out impact detection on the bottom of the Cabeus crater and other PSRs, and confirmed that there are volatiles such as water ice in the lunar soil released by the impacts [49].

Therefore, it is of great scientific value, as well as being beneficial for the development of applications, to carry out comprehensive scientific and resource exploration with the orbiter, lander, rover and mini-flying probe in the lunar south pole region, with communication support from the relay satellite. The scientific questions addressed by lunar polar missions can be categorized as follows.

### Distribution and origins of lunar water ice and volatile components

Water and volatiles on different celestial bodies provide not only important material records of their own formation and evolutionary process, but also key information about the evolution of the solar system [49,50]. Water ice and volatiles may exist in the lunar polar regions, but there is a lack of direct observational evidence. In 2009, the LCROSS, launched by the USA, intentionally crashed into the Moon's southern polar region and observed the evaporation of water and volatiles for the first time [51,52]. Subsequent precise geochemical analysis of Apollo samples also indicates that there is water (in the forms of H, OH and crystal structural H_2_O) on the Moon [51,53]. The inhomogeneity and complexity of the distribution of water in the interior and on the surface of the Moon are challenges for constraining the models of the origin and evolution of the Moon. Although some progress has been made in the study of water ice and volatiles in the lunar polar regions, there are still major scientific questions that remain to be answered, such as the abundance, distribution and isotopic compositions of lunar volatiles; the origins of volatiles; the migration, preservation, enrichment and depletion mechanisms of volatiles in PSRs; and the ancient solar wind environment.

Previous studies of water and volatiles were mainly based on orbital remote sensing neutron spectrometers [54–56], synthetic aperture radars [57–60], spectrometers [61–64] and other methods; only indirect evidence of the existence of water ice in the shadowed area was obtained, and it is difficult to judge its depth, abundance and forms, etc. By using more advanced high-precision neutron gamma spectrometers and synthetic aperture radars, and by conducting direct *in-situ* measurements of H_2_O molecules and their H isotopes in PSRs at the same time, we can not only confirm the existence of water ice and reveal its origin, but also obtain the distribution and content of water ice in the PSRs through a comparative analysis of *in-situ* measurement results with the remote sensing detection results of PSRs on the entire lunar surface. Combined with laboratory test analysis and research on water and volatile components in lunar samples, we may address fundamental questions on the origin and distribution of lunar water ice and volatile components.

### Lunar impact structure and impact history

The impacts of small celestial bodies were common and important events in the early solar system, and these impacts had a significant influence on the environment and evolution of planets, such as Earth and Mars. Due to long-term and intense geological effects, the early impact traces on Earth have been largely erased due to geological events, weathering processes and even human activities, whilst the Moon has well preserved the entire impact history of celestial bodies in the inner solar system [65,66]. The rocks and lunar soil in the SPA basin may have been transported from other regions during the impacts. By examining these materials, we could retrieve the impact history of the Moon and thus reveal the intensive impact events of terrestrial planets in the early solar system. Furthermore, volatiles exist in the PSRs of the lunar polar region, and their types, isotopic composition and spatial distribution can reveal early lunar collision processes. These will make significant contributions to the study of the composition of asteroids and comets that formed in the early solar system, the types of volatiles in the early lunar magma ocean and the degassing during crystallization, and the long-term lunar surface space environment [53,67,68].

Since many areas of the impact craters in the lunar south pole region are PSRs, there is little information about their topography and geological structures. This limits our understanding of the topography and structure of this area. High-precision remote sensing optical cameras and radars, as well as optical cameras and radars in the landing area, can be used to carry out joint *in-situ* and reconnaissance exploration to obtain full coverage of the morphology, structure, impact craters and impact crater distribution characteristics of the lunar south pole. Combined with the results of the material composition, the evolutionary history of the Moon's early topography, structure and impact history can be comprehensively reconstructed.

### Lunar deep materials and internal structure

The composition and internal structure of the Moon are core elements for understanding the origin and evolution of the Moon [69]. The compositions of the lower lunar crust and mantle are two of the key issues in lunar science, which are of great significance with regard to understanding the differentiation process in the early stages of lunar formation and the cause of the lunar dichotomy. The SPA basin is the oldest, largest and deepest impact basin on the Moon. It is possible that lunar mantle materials were excavated during the formation of the SPA basin, providing a natural geological profile for the study of the Moon's interior materials. The peak rings, impact melt layers in the basin and ejecta blankets are good candidate sources for lunar mantle materials. Although the excavated lunar mantle materials may be covered by crust-derived lunar soil and basalts, the impact craters that formed later can potentially re-expose the lunar mantle materials.

Due to the low illumination conditions in the polar region, it has been difficult for previous lunar remote sensing technology to effectively observe the small-scale material composition in this region. At present, the understanding of the material composition in this region mainly comes from neutron and gamma-ray spectra remote sensing technology with relatively low spatial resolution. Results of the material composition in this region via optical remote sensing technology are rare [42]. Using modern detectors to carry out precise *in-situ* and long-term continuous observations, combined with laboratory tests and analyses of the samples returned from the Moon, we can determine the compositions of the original lunar crust and mantle and the interior structure of the Moon, in order to reproduce the history and geological evolution of the lunar south pole region and thus reveal the processes of the compositional evolution of the Moon.

### Lunar surface space environment

The Moon does not have a dense atmosphere and global magnetic field like the Earth does, so the solar wind plasma, solar energetic particles, coronal mass ejection material, galactic cosmic rays, charged particles of the Earth's magnetotail and high-flux solar ultraviolet photons can directly reach and interact with the lunar surface, forming a complex environment of charged particles, electromagnetic fields and dust. Based on the space scale, the lunar plasma environment can be categorized into two types: large-scale and small-scale environments. The large-scale space environment includes the solar wind around the Moon, the Earth wind and the lunar wake formed by their interaction with the Moon [70]. The small-scale space environment includes the mini-magnetosphere [71] formed by the interaction between the solar wind and the local crustal remanence on the lunar surface and the plasma sheath [72] near the lunar surface, etc. At present, most of the data on the lunar mini-magnetosphere come from satellite observations, mainly the magnetosheath of the mini-magnetosphere. The internal structure of the mini-magnetosphere and the connection with the vortex phenomenon on the lunar surface are not clear, and more detailed observations are needed for further investigation.

It is necessary to obtain accurate characteristics of the lunar surface reflection/sputtering of charged particles, as well as the relationship between the lunar surface electric field and charged particle parameters, and build a lunar surface plasma sheath model in the lunar polar region by high-precision comprehensive particle, electromagnetic field and dust parameters. It is of high interest to evaluate the role of charged particles in the process of lunar soil weathering, the impact of lunar surface radiation on lunar surface facilities and astronauts, and to establish dynamic models of lunar dust in the polar region. Important issues also include verifying the causal relationship between the strong charging of the lunar surface caused by charged particles and the lunar dust fountain, and resolving the long-standing controversy about dust activity near the lunar terminator [73].

### Earth–Moon space environment

Earth–Moon space is a closely coupled system. The energy, momentum and mass output from the Sun, and their changes, influence the formation, structure and changes in the Earth**–**Moon space environment. The overall variation in the Earth**–**Moon space environment is a key factor in understanding the patterns of the Earth's space weather; therefore, imaging observations of the Earth's magnetosphere have received increasing attention. The phenomena currently known from imaging observations include the contraction of the plasmasphere and the generation of plasma plumes, the thinning and reconnection of the magnetotail current sheet during magnetic storms, and the enhancement and asymmetry of the Earth ring current. However, previous investigations on each physical layer or region have been relatively independent, and the dynamic characteristics, global distribution and causal relationship of various dynamical phenomena are still unsolved mysteries.

Based on the characteristics of plasma in different regions of the magnetosphere, different observational methods can be used to obtain images of specific regions. Images of the Earth's plasmasphere can be observed by an extreme ultraviolet imager [74], the ring current can be observed by the neutral atom imager in the high-energy band, and the magnetotail plasma sheet can be observed by the neutral atom imager in the middle-energy band [75]. By combining different images for different regions, we could address questions relating to coupling processes, such as how magnetotail reconnection and ring current enhancement during a magnetic storm affect the structure of the plasmasphere to produce plumes and whether plumes can reduce the energy input at the magnetopause as a whole to affect the process of magnetic storms.

## CANDIDATE LANDING SITE

**Figure 2.**
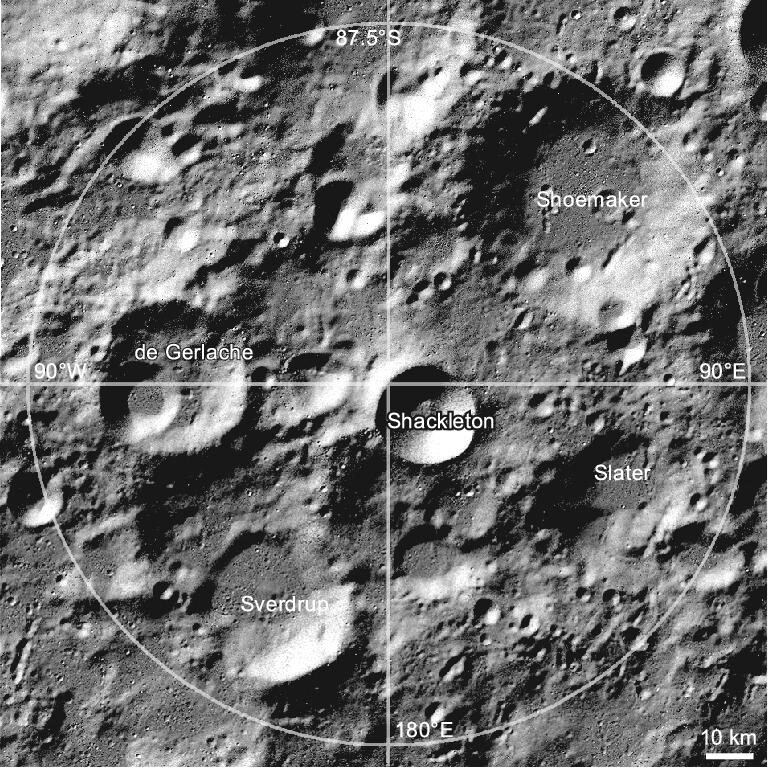
The candidate landing region of CE-7.

The preferred candidate landing site of the CE-7 probe is the region near Shackleton crater (Fig. 2), which is relatively geologically young and mainly composed of materials that formed during the Eratosthenian period (between 3.2 and 1.1 Ga) of the Moon [40,76,77]. Most areas outside Shackleton crater have ancient pre-Nectarian compositions [40], whose topographic features are primarily rugged highlands and widely distributed small craters, with only a few isolated and tiny smooth areas in the west [78,79]. Shackleton crater is located near the main rim of the SPA basin [40], and moonquakes have been detected on both the near and far sides. Furthermore, it is highly possible to collect deep lunar materials excavated by the SPA basin in the region, offering insights into the lunar internal structure and material composition [80,81].

Shackleton crater is a simple bowl-shaped crater with a diameter of 21 km and a depth of 4 km, whose average wall slope is as high as 30.5° [77]. Due to the Moon's low obliquity and the sharp rim of Shackleton crater, there exists a large PSR within Shackleton crater. Moreover, areas close to the rim receive ample sunlight [47,82], supporting the CE-7 lander with abundant solar energy.

Based on the measurements made by the Lunar Orbiter Laser Altimeter (LOLA) at a wavelength of 1064 nm [83], the Shackleton crater floor appears much brighter than the surrounding terrain and other craters. This could be attributed to the presence of a thin layer, estimated to be only 1 μm in thickness, that has a water ice content of 20% on the surface of the PSR within the floor [77]. Subsequent studies also support the existence of water ice in Shackleton crater [82,84,85]. Water is of very significant scientific interest and is also a key resource for future lunar development and utilization, with the potential to be used for oxygen and hydrogen production, washing and even drinking. The investigation of the content, distribution and isotopic composition of water ice in Shackleton crater could enhance the understanding of the source, transport, accumulation, sequestration, loss and other essential processes of volatiles during lunar evolutionary processes, helping to accurately estimate the reserves and storage status of the Moon's water. It is of great significance for our in-depth understanding of the distribution of water and volatiles on the Moon over time and space [26,86].

## SCIENTIFIC OBJECTIVES AND PAYLOADS OF CE-7

### Scientific objectives

To meet the scientific requirements and address the key scientific problems mentioned above, the CE-7 mission will focus on the lunar south pole region. The main scientific objectives include the following:

- Investigating water ice and volatile components in the lunar soil. CE-7 will help reveal the distribution and content of water ice on the lunar surface, especially in the PSRs, and carry out an analysis of volatile components of the lunar soil and an *in-situ* analysis of water ice, in an attempt to directly confirm the presence and origin of water ice on the Moon.
- Investigating the topography, composition and structure of the Moon. CE-7 will obtain high-precision topographic and compositional data, and construct a geological profile and map of topography, composition and subsurface structure in the south pole region, aiming to significantly contribute to the geological history and evolution of the region.
- Investigating the Moon's interior structure, magnetic fields and thermal characteristics. CE-7 will detect moonquakes, measure the magnetic field and obtain information on the Moon's deep interior structure and regional magnetic field gradient distribution to reveal the history of lunar evolution and the mechanism of the emerging and fading of the lunar magnetic field.
- Investigating the space environment of the lunar south pole. CE-7 will make observations on low-energy ions, medium-energy particles, high-energy particles, electromagnetic fields, lunar dust and magnetic field gradients and carry out comprehensive investigations of the environmental characteristics of the lunar south pole to reveal its interaction process and genetic mechanism and provide important environmental parameters for subsequent missions based on the lunar scientific research station.
- Investigating the Moon-based magnetotail and plasmasphere of the Earth. CE-7 will image Earth's magnetotail by way of energetic neutral atoms with high space-time and energy resolution to reveal the dynamics of the Earth's magnetotail and the mechanism of interaction between solar wind and Earth's magnetosphere and ionosphere.
- Supporting deep-space VLBI measurements and radio science by the Lunar Orbit VLBI Experiment (LOVEX), to improve the accuracy of orbit determination in deep space and to carry out radio astronomical observation.

### Scientific payloads

In order to achieve the above scientific objectives, CE-7 is equipped with a total of 18 scientific payloads. The relay satellite, orbiter, lander, rover and mini-flying probe carry 3, 5, 4, 5 and 1 payload, respectively.

**Figure 3.**
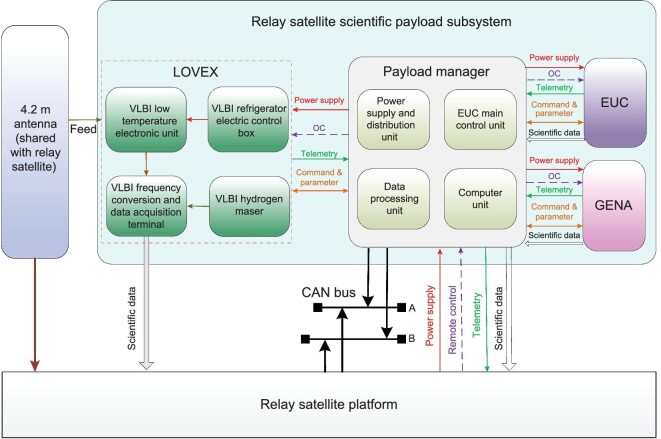
The relay satellite scientific payload subsystem.

The relay satellite is equipped with three scientific payloads, including the LOVEX, a grid-based energetic neural atom imager (GENA) and an extreme ultraviolet camera (EUC). To achieve resource optimization and sharing, a scientific payload manager is configured to provide unified power, telemetry, remote control, bus and scientific data interfaces for the scientific payloads onboard the relay satellite. The relay satellite scientific payload subsystem is shown in Fig. 3, and the main technical specifications are listed in Table 1.

**Table 1.** The main technical specifications of scientific payloads on the relay satellite platform.

Scientific payload	Specifications
LOVEX	Frequency: X (8–9 GHz) Band width: 64, 128, 256 and 512 MHz Gain: 28–62 dB Clutter suppression: ≤−50 dBc@0 dBm Harmonic suppression: ≤−35 dBc@0 dBm Noise factor: ≤3.5 dB
GENA	Neural atom species: H, O Energy range: 4–200 keV(H), 8–250 keV(O) Energy resolution: better than 1 keV@10 keV Fly time resolution: ≤1 ns Geometric factor: better than 14 cm^2^Sr Flux measurement sensitivity: better 3 counts/(cm^2^·s)
EUC	Double waveband central wavelength: 30.4 ± 0.5 nm, 83.4 ± 2.0 nm Spectral bandwidth: ≤5 nm@30.4 nm, ≤23 nm@83.4 nm Angular resolution: better than 0.3° Sensitivity: ≥0.4 counts/s/R/pixel @30.4 nm, ≥0.054 counts/s/R/pixel @83.4 nm Dynamic range: 0.01–15 R@30.4 nm, 0.1–300 R@83.4 nm Dark count rate: ≤1 counts/(cm^2^·s)

**Figure 4.**
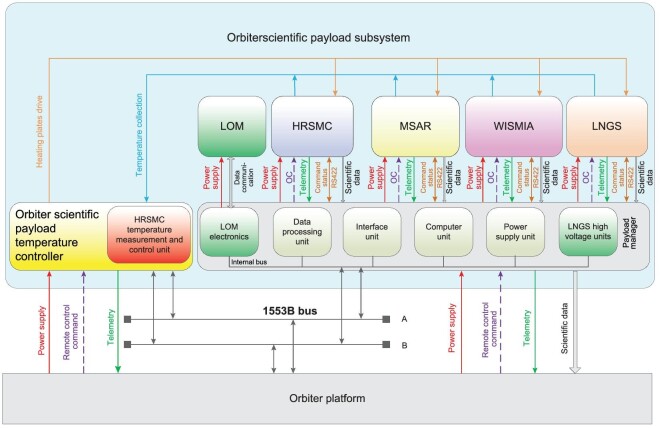
The orbiter scientific payload subsystem.

The orbiter is equipped with five scientific payloads, including a high-resolution stereo mapping camera (HRSMC), miniature synthetic aperture radar (MSAR), wide-band infrared spectrum mineral imaging analyzer (WISMIA), lunar neutron gamma spectrometer (LNGS) and lunar orbit magnetometer (LOM). Similar to the relay satellite, a scientific payload manager is also configured to provide unified power, telemetry, remote control, bus and scientific data interfaces for the orbiter scientific payloads. The LOM electronics and LNGS high-voltage units are integrated into the payload manager. The orbiter scientific payload subsystem is shown in Fig. 4, and the main technical specifications are listed in Table 2.

**Table 2.** The main technical specifications of scientific payloads on the orbiter.

Scientific payload	Specifications
HRSMC	Resolution and width: Panchromatic resolution is better than 0.5 m, width ≥ 18 km@orbit altitude 100 km Panchromatic resolution is better than 0.075 m, width ≥ 0.9 km@orbit altitude 15 km S/N: ≥50 dB (solar altitude of 80°, Albedo of 0.25, orbit altitude of 100 km); ≥35 dB (solar altitude of 0.6°, Albedo of 0.09, orbit altitude of 80 km); ≥34 dB (solar altitude of 1.5°, Albedo of 0.1, orbit altitude of 15 km); ≥27 dB (solar altitude of 0.6°, Albedo of 0.09, orbit altitude of 15 km); Base-height ratio: ≥0.5 Spectrum: 0.45 μm –0.90 μm MTF: ≥0.15@Nyquist Distortion stability: ≤0.3 pixel (half year)
MSAR	Resolution: better than 1 m Width: 5 km–20 km Polarization: multi-polarization FOV: 15°–45°
WISMIA	Spectral range: 0.45 μm–10.0 μm Spectral resolution: ≤10 nm@0.45–3.0 μm, ≤200 nm@3.0–10.0 μm Spatial resolution: ≤0.2 mrad@0.45–3.0 μm, ≤0.3 mrad@3.0–10.0 μm FOV: ≥3.8° MTF > 0.1
LNGS	Neutron: Thermal neutron energy: 0–0.4 eV Epithermal neutron energy: 0.4 eV–700 keV Fast neutron energy: 700 keV–5 MeV Gamma spectrometer: 0.3–9 MeV
LOM	Measuring range: ±2000 nT Stability: ≤0.01 nT/°C Resolution: better than 0.01 nT

**Figure 5.**
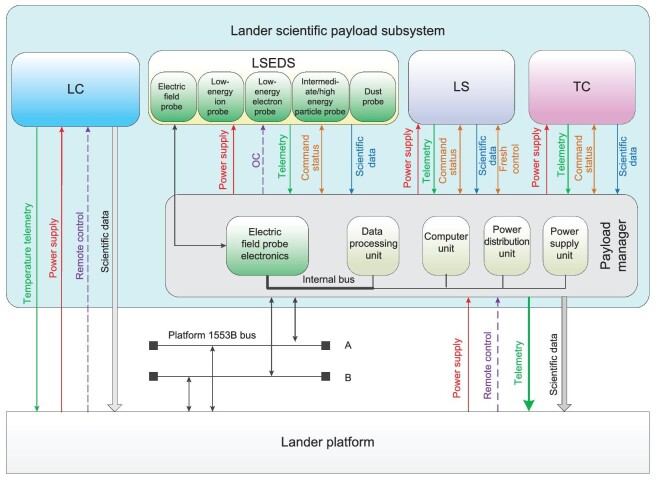
The lander scientific payload subsystem.

The lander is equipped with four scientific payloads, including a lunar seismograph (LS), lunar surface environment detection system (LSEDS), landing camera (LC) and topography camera (TC). A lander scientific payload manager is also configured in the same way as the orbiter and the relay satellite. The LC directly interacts with the lander platform and the RM, the LRS and the LPR are based on the rover scientific payload manager to exchange information with the lander platform via the lander scientific payload manager to exchange information. The lander scientific payload subsystem is shown in Fig. 5, and the main technical specifications are listed in Table 3.

**Table 3.** The main technical specifications of scientific payloads on the lander.

Scientific payload	Specifications
LC	Waveband: visible spectrum Imaging distance: 4 m–∞ FOV: 45° × 45° MTF: ≥0.20 S/N (dB): ≥30 (Albedo ≥ 0.09, solar altitude ≥ 0.6°)
TC	Waveband: visible spectrum FOV: 17.4° × 17.4° S/N (dB): ≥30 (Albedo ≥ 0.09, solar altitude ≥−0.30°) MTF: ≥0.2
LS	Frequency band range: 1/120–100 Hz Dynamic range: >120 dB Measuring range: −4–+4
LSEDS	Particle energy range: Electronic: 1 eV–12 MeV, proton: 1 eV–300 MeV, heavy ion: 8 MeV–300 MeV/n Particle flux range: 10^7^@1 eV–30 keV, 10^5^@30 keV–300 MeV/n Particle radiation effect: Sensitivity: 20 μrad(Si)/h LET spectrum: 0.001–37 MeV/(mg/cm^2^) Electromagnetic field sensitivity: Electronic field 1 μV/m@±10 V/m, Magnetic field: 3 pT@±1024 nT, 50 pT@±65000 nT Accumulated dust mass: 1 × 10^−9^–3 × 10^−4^ g/cm^2^@Triorthogonal direction Dust particle size: 1 μm–5 mm

**Figure 6.**
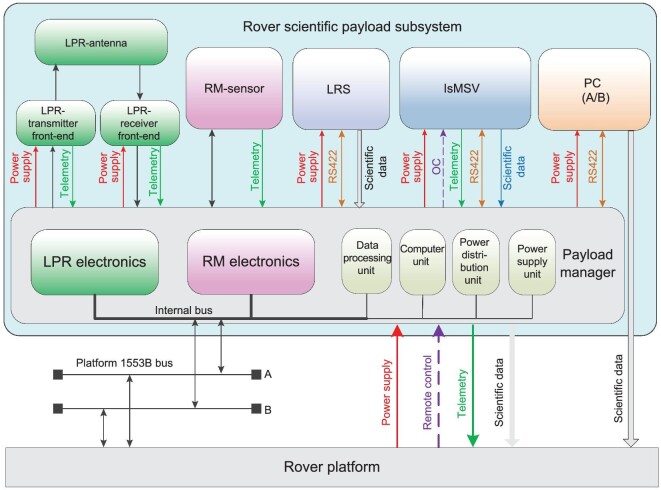
The rover scientific payload subsystem.

The rover is equipped with five scientific payloads, including a panoramic camera (PC), rover magnetometer (RM), the lunar raman spectrometer (LRS), the lunar penetrating radar (LPR) and the *in-situ* measuring system of volatiles on the lunar surface (IsMSV). A rover scientific payload manager is configured to provide unified power, telemetry, remote control, bus and scientific data interfaces for the rover scientific payloads. The PC directly interacts with the lander platform, and the RM, the LRS and the LPR are based on the rover scientific payload manager to exchange information with the lander platform. The rover scientific payload subsystem is shown in Fig. 6, and the main technical specifications are listed in Table 4.

**Table 4.** The main technical specifications of scientific payloads on the rover.

Scientific payload	Specifications
PC	Waveband: visible spectrum FOV: ≥16° × 16° S/N: ≥30 (Albedo ≥ 0.09, solar altitude ≥ −0.30°) MTF: ≥0.2
RM	Measuring range: ±2000 nT Magnetometer stability: ≤0.01 nT/°C Resolution: better than 5 pT
LRS	Spectrum range: 300–6000 cm^−1^ Spectrum resolution: better than 10 cm^−1^ Microscopic imaging pixel: ≥256 × 256
LPR	Frequency: 100 MHz–1500 MHz, 10 MHz–110 MHz Depth resolution: better than 15 cm@100 MHz–1500 MHz, and better than 2 m@10 MHz–110 MHz Depth: ≥40 m@100 MHz–1500 MHz, ≥400 m@10 MHz–110 MHz Polarization: multi-polarization
IsMSV	Analysis range: 2–150 amu Mass resolution: ≤1 amu Volatiles type: H_2_, He, H_2_O, Ne, N_2_, CO, CO_2_, Ar, Xe, NH_3_, CH_4_, C_2_H_6_ Mass spectrometer precision@(10^−5^ Pa partial pressure Ar): better than 1% Volatiles contents detection limit: better than 0.01 wt%

**Figure 7.**
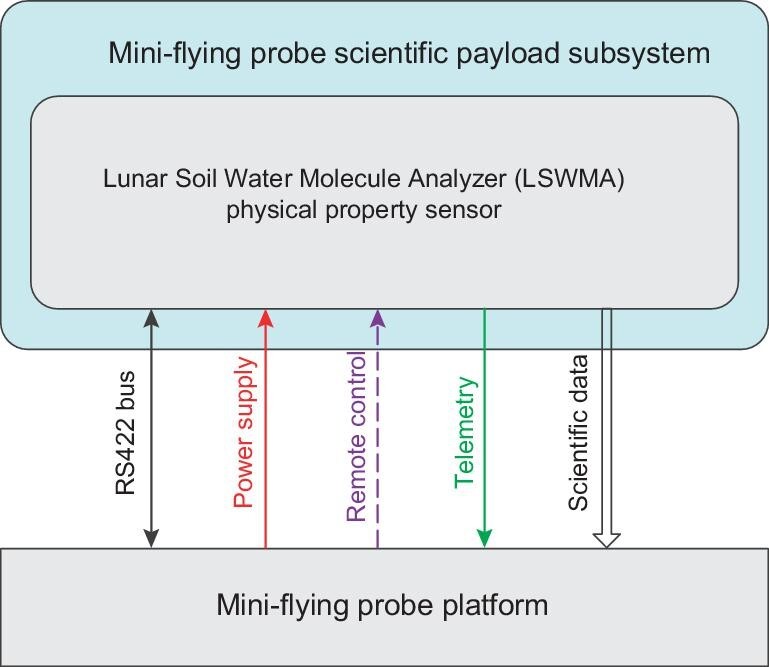
The connection between the LSWMA and the mini-flying probe platform.

The mini-flying probe is equipped with one scientific payload, a lunar soil water molecule analyzer (LSWMA). The LSWMA is composed of a gas acquisition unit, a mass spectrometer unit, a spectrum unit, an electronics unit, a pipeline assembly and a physical property sensor. The LSWMA directly interacts with the mini-flying probe platform. The connection between the LSWMA and the mini-flying probe platform is shown in Fig. 7, and the main technical specifications are listed in Box 1.

**Box 1.** The main technical specifications of scientific payloads on the mini-flying probe platform.

Scientific payload	Specifications
LSWMA	H_2_O detection limit: better than 0.01 wt% Measuring error: better than 50% Detectability: mass number <100 amu H_2_O S/N: better than 1000 Analytical quantity: ≥30 times

### Main scientific tasks

To detect the distribution and content of water ice on the lunar surface, especially in the PSRs, the LGNS is installed on the orbiter. The abundance of water molecules and hydrogen isotopes are obtained by using the LSWMA. The data are then used to directly confirm the existence and source of water ice in the PSRs at the lunar south pole. The noble gas and isotope data of the lunar soil are obtained by using the IsMSV on the rover, and the content and distribution of noble gas resources in the lunar soil are evaluated.

To investigate the topography, composition and structure of the Moon, the HRSMC and MSAR on the orbiter are used to obtain the topography of the full surface and the PSRs of the Moon. The topography and subsurface structure are obtained by means of the LC, TC, PC and LPR. The contents and distribution of elements and minerals on the lunar surface are detected by the LNGS and WISMIA on the orbiter.

To measure the internal structure, magnetic field and thermal characteristics of the Moon, two magnetometers installed on the orbiter and rover are used to obtain the magnetic field characteristics and regional distribution gradient characteristics of the Moon. The thermal anomaly data of the lunar surface are obtained by using the thermal infrared band of the WISMIA on the orbiter. The LS on the lander is used to obtain lunar seismometer data and to study the interior structure of the Moon.

The LSEDS and RM are used to obtain environmental data, which can be studied to interpret the causal mechanism of magnetic anomalies in the lunar vortex area and the abnormal distribution of lunar dust in time and space, to reveal the comprehensive environmental characteristics and interaction mechanism of the lunar surface and to provide important environmental parameters for subsequent lunar scientific research station missions.

To carry out Moon-based observation of Earth's magnetotail and plasmasphere, GENA, which is on the satellite, can obtain energetic neutral atom imaging data of Earth's magnetotail with high spatiotemporal and energy resolution. Using the EUC on the relay satellite, the morphology of the Earth's plasmasphere and the distribution characteristics of helium and oxygen ions in near-Earth space are obtained. The comprehensive characteristics of the Earth's magnetotail and plasmasphere in different periods of solar activity are studied to reveal the interaction mechanism between the solar wind, the Earth's magnetosphere and the ionosphere.

The LOVEX on the relay satellite is used to construct a 400 000-km baseline Moon–Earth VLBI measurement and observation experiment system to improve the accuracy of orbit determination in deep space and to carry out astrometry and astrophysics observation and study.

The associations between scientific payloads and scientific tasks are shown in Table 5. It is important to remember that the scientific payloads and tasks are subject to minor adjustments due to engineering constraints.

**Table 5.** Scientific objectives of the CE-7 mission.

		Scientific payloads				
Scientific objectives	Scientific tasks	Orbiter	Lander	Rover	Mini-flying probe	Relay satellite
Study on the distribution and source of lunar water and volatiles, directly confirming the presence and source of water ice on the Moon.	Obtaining the distribution and source of lunar water ice in the lunar south pole and permanently shadowed area. Obtaining the water molecule and hydrogen isotope data of the flyer landing site.	LNGS			LSWMA	
	Obtaining the rare gas and isotope detection data in the lunar regolith.			IsMSV		
Study on lunar morphology, composition and structure.	Obtaining the topographic data of the lunar south pole and PSRs.	HRSMC				
		MSAR				
	Obtaining the topographic and subsurface structural data.		LC	PC		
			TC	LPR		
	Obtaining the distribution and content of elements and minerals on the lunar surface.	WISMIA				
		LNGS				
	Obtaining the mineral component data.			LRS		
Study on lunar crust–mantle–core structures, mineral and element components, and the characteristics of the electric and magnetic fields, heat flow and gravitational field.	Obtaining the characteristics of the lunar magnetic field and regional distribution gradient.	LOM		RM		
	Obtaining the thermal anomaly data.	WISMIA				
	Obtaining the lunar moonquake data and researching the crust–mantle–core structures.		LS			
Study on the space environment such as the lunar surface magnetic field, lunar dust and particle radiation, and revealing the mechanism of solar wind causing the magnetic anomalies in the vortex region of the lunar surface.	Obtaining the environmental data of the lunar surface.		LSEDS	RM		
Study on the Moon-based observations of Earth's magnetotail and plasma layer.	Imaging via the energetic neutral atom imager with high spatiotemporal and energy resolutions of the Earth's magnetic tail.					GENA
	Obtaining the morphology of the plasma layer and the distribution characteristics of helium and oxygen ions in near-Earth space.					EUC
Study on the baseline Moon–Earth VLBI measurement and observation experiment.	Constructing a Moon-based Earth VLBI measurement and observation experiment system with a 400 000 km baseline.					LOVEX

## SUMMARY

Lunar exploration is crucial to understanding the Earth–Moon system and solar system. After successful implementation of the three phases, i.e. ‘orbiting, landing and returning’, the CLEP in China has initiated the development of the fourth phase of the lunar exploration project.

As the cornerstone mission of the fourth phase of the lunar exploration project, the CE-7 mission will focus on the lunar south pole, equipped with 18 sets of scientific payloads. It is composed of four modules, namely orbiting, landing, roving and mini-flying modules, to conduct scientific and resource investigations in the lunar south pole. It is expected to achieve major original scientific advances with regard to the formation and evolution of the Moon, the Sun–Earth–Moon space environment, and the *in-situ* utilization of lunar resources, laying a foundation for the establishment of a long-term, continuously operating comprehensive lunar scientific research station in the future.
